# Purification and Characterization of a Novel Calcium-Binding Heptapeptide from the Hydrolysate of Tilapia Bone with Its Osteogenic Activity

**DOI:** 10.3390/foods11030468

**Published:** 2022-02-04

**Authors:** Jinlun He, Hao Guo, Mei Zhang, Meng Wang, Liping Sun, Yongliang Zhuang

**Affiliations:** Faculty of Food Science and Engineering, Kunming University of Science and Technology, No. 727 South Jingming Road, Kunming 650500, China; hejinlun00@163.com (J.H.); guohao3343566283@163.com (H.G.); ynmzhang@163.com (M.Z.); wmjpl0220@163.com (M.W.); lpsun@kmust.edu.cn (L.S.)

**Keywords:** tilapia bone, peptide, calcium-binding capacity, structural characteristics, mass spectrometry, molecular docking, osteoblast

## Abstract

In this study, a calcium-binding peptide was obtained by hydrolyzing tilapia bone and its osteogenic activity was evaluated. Animal protease was selected from nine enzymes, and its hydrolysate was purified through preparative and semi-preparative reverse phase high-performance liquid chromatography. The purified peptide was identified as DGPSGPK (656.32 Da) and its calcium-binding capacity reached 111.98 µg/mg. The peptide calcium chelate (DGPSGPK-Ca) was obtained, and its structure was characterized through Fourier transform infrared spectroscopy (FTIR), X-ray diffraction (XRD), scanning electron microscopy (SEM), and mass spectrometry (MS). The results of XRD and SEM showed that DGPSGPK-Ca was formed as a new compound. The carboxyl and amino groups of Lys and Asp residues may be the chelating sites of DGPSGPK according to the FTIR and MS results. The molecular simulation showed the carbonyl groups of Asp, Pro, Ser, and Lys residues involved in the binding of calcium. The interaction of DGPSGPK and different integrins was evaluated by molecular docking simulation, and the main forces involved were electrostatic interaction forces, hydrogen bonding and hydrophobic interactions. Furthermore, DGPSGPK could inhibit the differentiation of osteoclast and promote the proliferation, differentiation and mineralization of osteoblasts.

## 1. Introduction

Calcium is a crucial nutrient for human health and one of the most important inorganic elements in the human body [1]. In the form of hydroxyapatite crystals, calcium plays an important function in bones [2]. As the most prevalent inorganic element in the human body, calcium is engaged in a variety of biological activities related to whole-body metabolism, such as skeletal support, muscle contraction, and heart function [3]. Calcium deficiency can cause rickets, osteoporosis, osteomalacia, and other serious metabolic disorders [4]. A great variety of calcium products have been developed in recent years, which have helped alleviate health concerns caused by calcium shortage to some extent [5]. However, most calcium supplements offer an appropriate quantity of calcium and neglect the absorption process of calcium [6]. For this reason, effective calcium supplements have attracted considerable interest, and many studies have focused on discovering good calcium carriers [7].

Osteoporosis is a systemic disease that is generally divided into primary and secondary diseases [8]. Osteoporosis is generally caused by an imbalance between bone formation by osteoblasts and bone resorption by osteoclasts [9]. Osteoblasts are the material basis of osteogenesis and bone formation. Osteoblasts are also the most important functional cells in the continuous bone renewal activities [10]. In bone reconstruction, osteoblasts regulate bone mineralization by secreting bone matrix and non-collagenous proteins, and osteoclasts are directly involved in the bone resorption process [11]. Integrins are transmembrane receptors that can mediate the connection between the cell and the external environment. Integrins are heterodimeric subunits of α and β linked by non-covalent bonds [12]. A previous study indicated that some proteins and peptides can bind to integrin α5β1 to increase the adhesion of osteoblasts to promote osteoblast proliferation and mineralization [13]. Meanwhile, some proteins and peptides can bind to integrin αvβ3 to inhibit the proliferation and differentiation of osteoclasts [14].

Peptides have higher absorptivity, lower energy consumption and carrier saturation, compared with protein and free amino acids [15]. Some food-derived peptides have potential activities for calcium binding and improve the biological potency of calcium ions. Chen et al. isolated and purified a peptide (Asp-Gly-Asp-Asp-Gly-Glu-Ala-Gly-Lys-Ile-Gly) with high calcium binding capacity from tilapia scales and found that the complex between this peptide and calcium can be used as a calcium supplement for calcium-deficient rats [16]. Charoenphun et al. hydrolyzed a tilapia fish protein and obtained a short peptide (WEWLHYW) with a calcium binding capacity of 65 µg/mg [17]. The calcium binding capacity of EG reached 67.81 µg/mg. Liu et al. identified and characterized calcium-binding peptides in tilapia skin gelatins [18]. Huang et al. purified a peptide from whey protein hydrolysate and identified it as Glu-Gly (EG) [19]. Xu et al. isolated several peptides from *Mytilus edulis* with osteogenic activity [20].

The global market demand for tilapia (*Oreochromis niloticus*) is gradually increasing [21]. Tilapia is an important economic fish in China, and the tilapia fish processing industry generates a variety of by-products, including bone, skeleton, skin, and head, which have high utilization values [22]. Chuesiang and Sanguandeekul obtained a protein hydrolysate from tilapia bone and evaluated its antioxidant and angiotensin-I-converting enzyme (ACE)-inhibitory activities [23]. Lin et al. studied the preparation, purification, and identification of iron-chelating peptides derived from tilapia skin collagen and characterized peptide–iron complexes [22]. Chen et al. isolated a calcium-binding peptide, DGDDGEAGKIG, from tilapia scale protein hydrolysate and examined its calcium bioavailability in rats [16]. Liao et al. obtained three calcium-chelating peptides from tilapia bone collagen hydrolysate with amino acid sequences being GPAGPHGPVG, FDHIVY, and YQEPVIAPKL, respectively [24]. Our previous research revealed that tilapia skin hydrolysates exhibited strong antioxidant, anti-photoaging, and ACE inhibitory properties [25,26,27].

In this study, nine enzymes were used to degrade tilapia bone for the preparation of hydrolysates with calcium binding capacities. The key peptide in the hydrolysate was purified and identified, and the peptide calcium chelate was prepared. Then, the purified peptide and its peptide calcium chelate were characterized through Fourier transform infrared spectroscopy (FTIR), X-ray diffraction (XRD), scanning electron microscopy (SEM), mass spectrometry (MS), and molecular simulation. Furthermore, the osteogenic activity of the purified peptide was evaluated using osteoclasts and osteoblast culture in vitro. This study potentially provides the theoretical groundwork for calcium supplements utilizing tilapia bone-derived calcium binding peptides.

## 2. Results and Discussion

### 2.1. Different Degrees of Hydrolysis (DH) and Calcium Binding Capacities of Different Hydrolysates

Enzymatic hydrolysis is an effective method for modifying proteins, particularly improving the digestibility of proteins, reducing allergy, and producing peptides with high functions [28]. DH reflects the percentage of cleaved peptide bonds relative to the number of original peptide bonds in the protein [29]. For the production of a peptide with excellent calcium binding capacity, nine proteases were used to enzymatically hydrolyze tilapia bone. These enzymes are frequently used in preparing protein hydrolyses.

**Figure 1:**
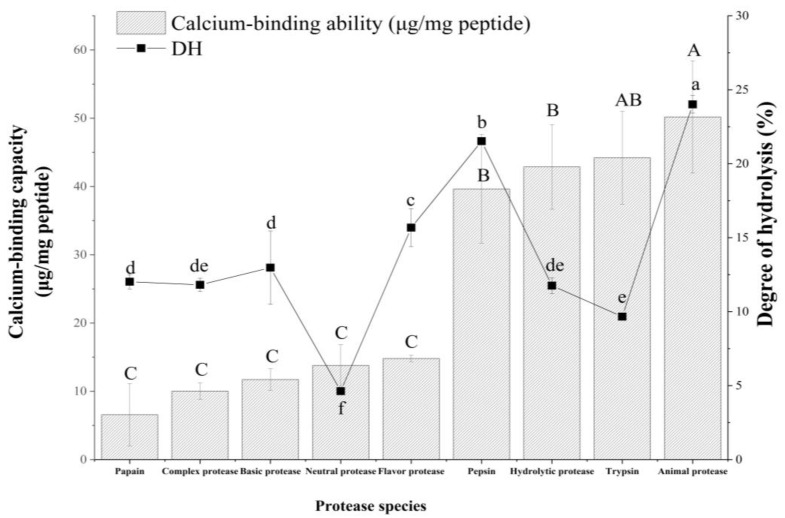
DH values and calcium binding capacities of nine hydrolysates from tilapia bone. Different lowercase letters demonstrated a significant difference in DH values (*p* < 0.05), and different capital letters indicated a significant difference in calcium binding capacities (*p* < 0.05).

As shown in Figure 1, the hydrolysate of animal protease had a higher DH and calcium binding capacity. The DH reached 24.01%, and the calcium chelating capacity reached 50.18 µg/mg. Animal protease is an endo- and exopeptidase with a wide range of sites compared with other enzymes. Animal protease hydrolysates have high calcium binding capacities because enzymatic hydrolysis sites provide short peptides. Increases in short peptides lead to an increase in calcium-binding sites that improve calcium binding capacity. Therefore, the enzymatic hydrolysate of animal protease (TBEH) was selected for subsequent experiments.

### 2.2. Separation and Purification of Calcium-Chelating Peptides

**Figure 2:**
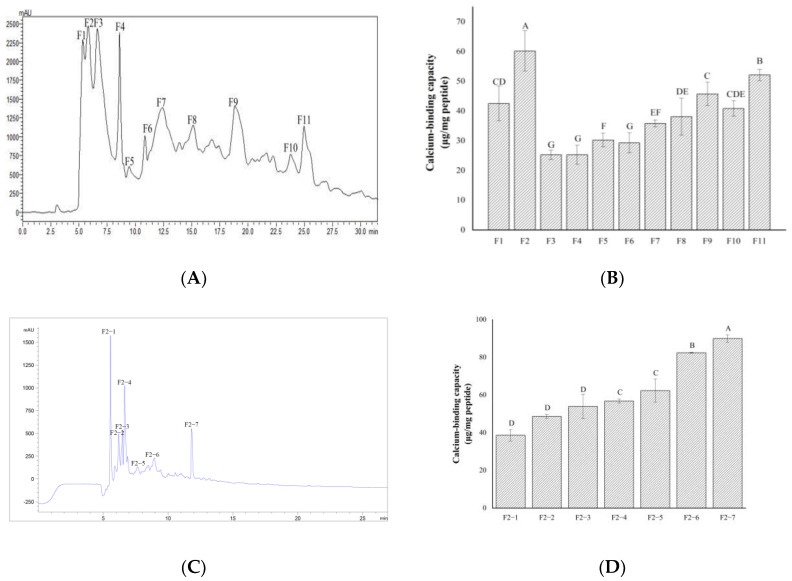
Elution curves of tilapia bone hydrolysates and calcium binding capacity of each fraction. (**A**) Shimadzu C−18 separated components. (**B**) Calcium binding capacity of Shimadzu C−18 separated components. (**C**) ZORBAX SB−C18 separated components. (**D**) Calcium binding capacity of ZORBAX SB−C18 separated components.

Preparative HPLC has the advantages of fast separation efficiency and large preparation quantity [30]. A preparative SHIMADZU C-18 column was used for the first separation step. As shown in Figure 2A, TBEH was divided into 11 fractions, which were named F1–F11. The calcium binding capacities of the fractions were determined. As shown in Figure 2B, the calcium binding capacity of F2 reached 60.18 µg/mg, which was significantly higher than the calcium binding capacities of the other fractions. Therefore, F2 was further separated using a semi-preparative column (ZORBAX SB-C18). Seven fractions (F2-1–F2-7) were collected as shown in Figure 2C, and their calcium-binding capacities were evaluated. As shown in Figure 2D, F2-7 had the strongest calcium binding capacity.

### 2.3. Amino Acid Sequence Identification

**Figure 3:**
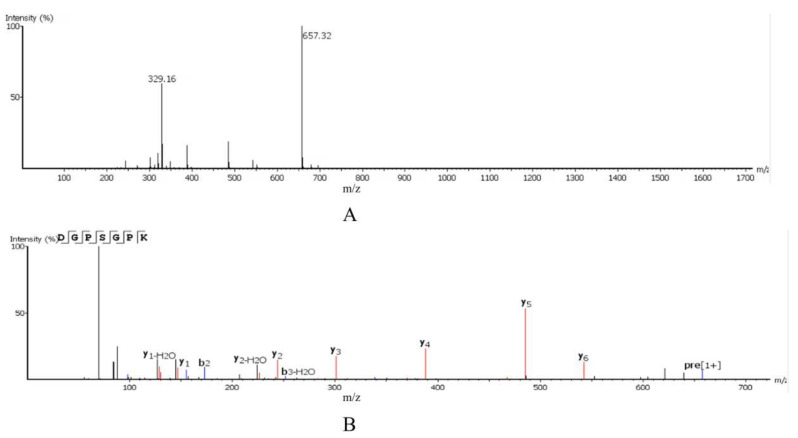
MS/MS spectrum of DGPSGPK. (**A**) Primary mass spectrum; (**B**) secondary mass spectrum.

F2-7 was further analyzed using UPLC-Q-Orbitrap-MS^2^, and the amino acid sequence was confirmed with *De Novo ^TM^* (Peak Studio 7.5, BioInfor-Matics Solutions, Inc. Waterloo, ON, Canada). As shown in Figure 3A, molecular ion peak [M+H]^+^ and double charge molecular ion peaks [M+2H]^2+^ were identified at m/z values of 657.32 and 329.16 Da. According to the ratio of mass to charge, the molecular weight of the peptide purified was 656.32 Da. As shown in Figure 3B, the peptide fragment was cleaved into Y-type (N-terminal) and B-type (C-terminal) fragments. The peptide’s amino acid sequence was DGPSGPK. Subsequently, the peptide was synthesized. The calcium binding capacity of DGPSGPK was determined, being 111.98 µg/mg.

DGPSGPK had two “Gly-Pro-X” sequences, which were the basic characteristics of collagen peptide. The continuous repeating “Gly-Pro-X” and “Gly-X-Y” sequences play crucial roles in stabilizing intra- and interchain hydrogen bonding in collagen structures [31]. Collagen peptides can effectively promote calcium binding capacity, such as GPAGPHGPVG [32], AGAAGEAGKIG [24], and GDKGESGEAGER [33]. Furthermore, Asp and Glu provide favorable environments for calcium-binding peptides [34], such as DGDDGEAGKIG isolated from tilapia scale protein hydrolysate and NDEELNK from sea cucumber ovum [16,30]. In addition, the sequence of the peptide, which contained Asp at the beginning and Lys at the end, showed high calcium-chelating capacity, such as DEEENDQVK [7]. Owing to the hydroxyl group on the side chain, Ser residue is a significant contributor to calcium-chelating capacity [35].

### 2.4. Structural Characterization

The structures of DGPSGPK and DGPSGPK-Ca were further characterized through FTIR, XRD, SEM, and UPLC-Q-Orbitrap-MS^2^, and the possible molecular mode of DGPSGPK-Ca was predicted.

#### 2.4.1. FTIR

**Figure 4:**
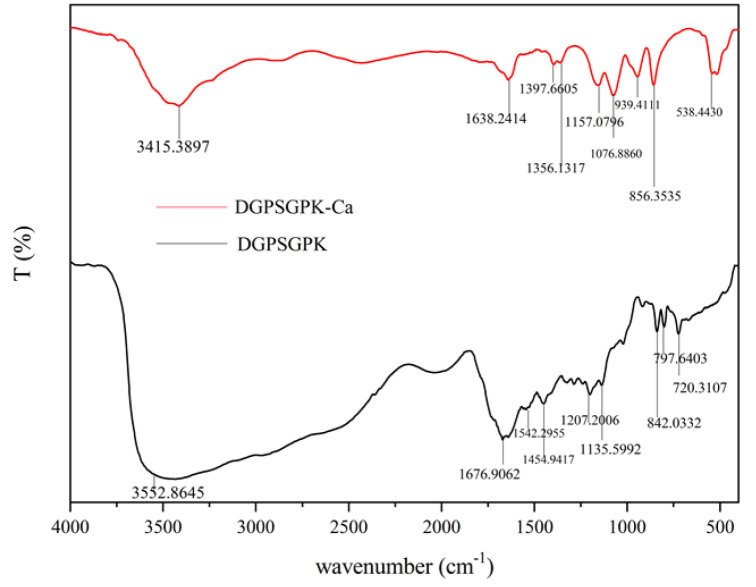
Infrared scanning spectrum of DGPSGPK and DGPSGPK−Ca.

The FTIR spectra of DGPSGPK and DGPSGPK-Ca are shown in Figure 4. The peak in the typical area was attributed to the N-H stretching vibration at 3485 cm^−1^ [36]. Meanwhile, the two most significant vibrational modes of amides in the fingerprint region are amide-I (1700–1600 cm^−1^) and amide-II (1600–1500 cm^−1^). The stretching vibration of C=O generated the amide-I vibration at 1676 cm^−1^ [37], and the stretching vibration of C–N and the bending vibration of N–H caused the amide-II vibration at 1543 cm^−1^ [38]. The stretching vibration of C–O generated the absorption peak at 1135 cm^−1^ [39]. The stretching vibration of O=C–NH generated the absorption peak at 842 cm^−1^ [40]. The absorption peak of N-H stretching vibration was shifted to 3422 cm^−1^ after DGPSGPK bonded with calcium ions, suggesting that the dipole field effect or inductive effect caused the electron cloud density of N-H in DGPSGPK to decrease [41]. The absorption peak of C=O in amide-I changed to 1638 cm^−1^, whereas the absorption peak of N-H in amide-II shifted to 1566 cm^−1^, suggesting that the amide carbonyl (C=O) and N–H of the amide bond contributed to the binding process between the peptide and calcium ion. Furthermore, the peak at 842 cm^−1^ in DGPSGPK shifted to 939 cm^−1^ in DGPSGPK-Ca because of the rise in O=C-NH electron cloud density and reduction in N-H electron cloud density during chelation [8]. FTIR results indicated that calcium mainly interacted with carboxylic oxygen and amino nitrogen atoms binding to DGPSGPK, which is in accordance with previous studies [8,38,40].

#### 2.4.2. XRD

**Figure 5:**
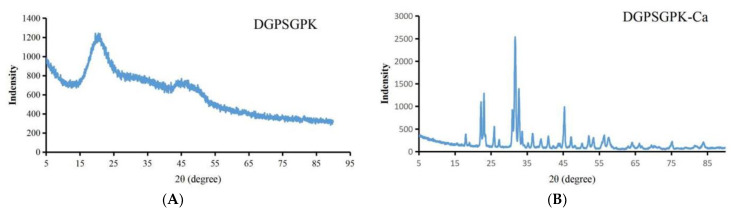
X-ray diffraction pattern of DGPSGPK (**A**) and DGPSGPK-Ca (**B**).

XRD is always used for evaluating morphological changes in molecular crystals. As shown in Figure 5A, DGPSGPK has a disordered arrangement and weak intensity at approximately 20°. The amino acids of the peptide were irregular and amorphous and had no absorption peak. However, as shown in Figure 5B, when DGPSGPK bonded with calcium, clear sharp peaks appeared at 22°, 31°, and 45.3°, and many small and sharp peaks formed. This result indicated that the crystal morphology of the calcium–peptide chelate changed and a new crystal morphology formed [42].

#### 2.4.3. SEM

**Figure 6:**
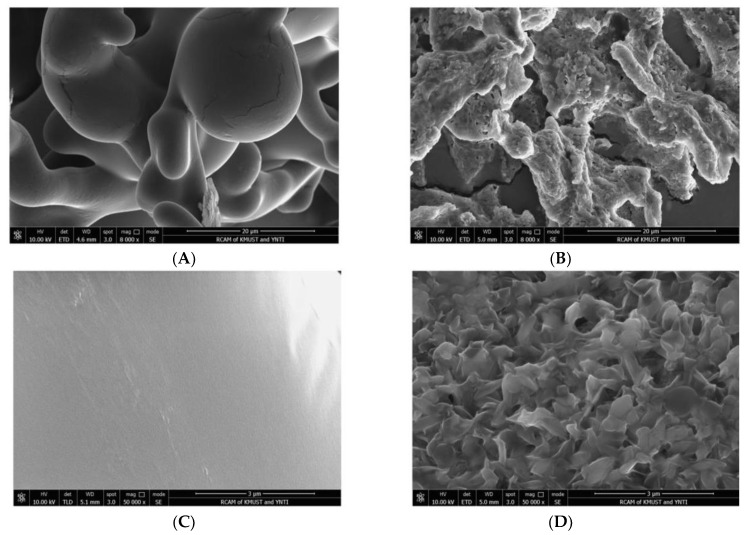
Scanning electron microscope images of DGPSGPK and DGPSGPK-Ca. (**A**) DGPSGPK at ×8000 magnification, (**B**) DGPSGPK-Ca at ×8000 magnification, (**C**) DGPSGPK at ×50,000 magnification, (**D**) DGPSGPK-Ca at ×50,000 magnification.

SEM can directly observe the surface structures of DGPSGPK and DGPSGPK-Ca. The SEM results of DGPSGPK are shown in Figure 6A,C. The surface of DGPSGPK was smooth and uniform, and some slight cracks appeared, which may have been generated during the rapid vacuum freeze-drying of the peptide [43]. As shown in Figure 6B,D, the surface of DGPSGPK-Ca was extremely rough and had small granular objects. The combination of the peptide and calcium may have caused these structures to change [18].

#### 2.4.4. UPLC-Q-Orbitrap-MS^2^

**Figure 7:**
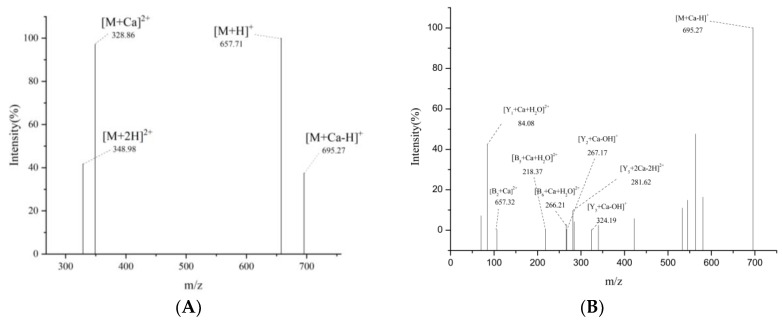
Identification of the binding sites of DGPSGPK-Ca. (**A**) Primary mass spectrum; (**B**) secondary mass spectrum.

The mass change of calcium chelated peptides is an excellent method to research how metal ions interact with organic ligand groups in peptides [44]. As can be seen from Figure 7A, the m/z values corresponding to the mother ions [M+Ca]^2+^ and [M+Ca-H]^+^ of DGPSGPK-Ca were 328.56 and 695.27 Da, respectively. The parent ion fragments were analyzed for the determination of the calcium-chelating sites.

The precursor ion [M+Ca-H]^+^ was used in examining the small peptide fragments in Figure 7B. The results indicated that DGPSGPK combined with calcium to form a new complex. It was then decomposed into multiple fragments for MS/MS analysis. The structural information provided by the fragmented ions contributed to the analysis of calcium ion binding sites. Fragment ions with m/z values of 106.74, 218.37, and 266.21 Da represented the peak of [B_2_+Ca]^2+^, [B_5_+Ca+H_2_O]^2+^, and [B_6_+Ca+H_2_O]^2+^, respectively. The calcium binding site might be related to Asp-Gly-, Asp-Gly-Pro-Ser-Gly-, and Asp-Gly-Pro-Ser-Gly-Pro-. This result indicated that the N-terminal of Asp of the peptide can bind to calcium. As is shown in Figure 7B, [Y_1_+Ca-H_2_O]^2+^ was the most abundant ion fragment, and its corresponding m/z was 84.08 Da. The results showed that calcium bound to the C-terminal of Lys of the peptide. [Y_2_+Ca-OH]^+^ and [Y_5_+2Ca-2H]^2+^ were observed at m/z values of 267.08 and 281.62 Da, indicating that Lys-Pro- and Lys-Pro-Gly-Ser-Pro- bound to Ca^2+^. According to FTIR and UPLC-Q-Orbitrap-MS^2^ analysis, the carboxyl and amino groups of Asp and Lys residues are mainly involved in the chelating site of Ca^2+^.

#### 2.4.5. Construction of the Possible Molecular Modes

**Figure 8:**
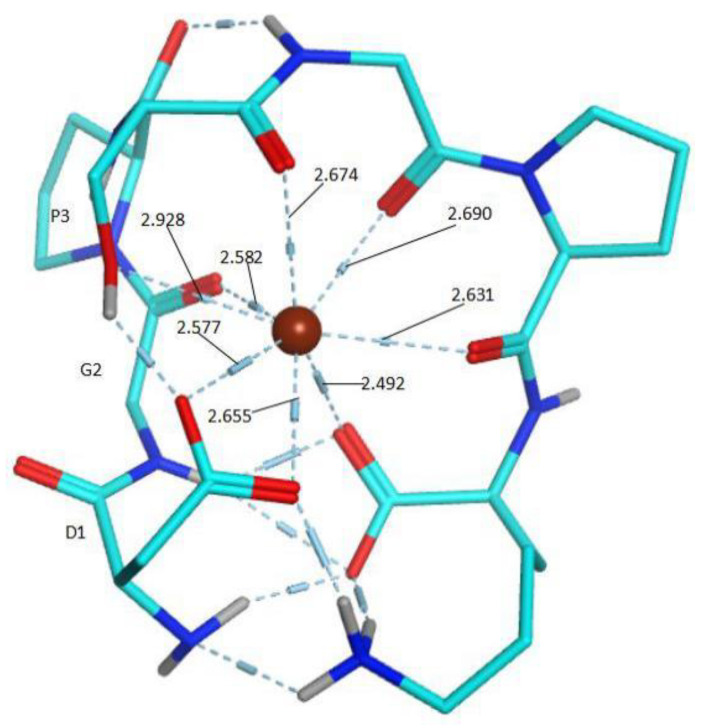
Snapshots of molecular dynamic simulation of DGPSGPK-Ca.

PEP-FPLD is a method for understanding the exact conformations of protein fragments [45]. The initial structure of the peptide was obtained through PEP-FPLD, and molecular simulation was carried out using the Charmm36 force field and TIP3P model. As shown in Figure 8, the simulation results showed that the chelating effect of peptide on Ca^2+^ primarily involves ionic bonding and covalent bonding formed by the carboxyl oxygen atoms of Asp, Pro, Ser, and Lys residues to Ca^2+^. The bond length of Ca-O was in the range of 2.492–2.928 Å. According to the theory of hard and soft acids and bases [5,39], Ca^2+^ prefers oxygen atoms as ligands to form stable complexes, such as calmodulin. Therefore, the carboxyl oxygen atoms of amino acid residues in peptides can be coordinated to Ca^2+^.

### 2.5. Molecule Docking

Molecular docking is mainly a method used to study the molecular forces between ligands and receptors and to predict their affinity and binding modes [46]. In this study, SYBYL-X 2.0 software was used to evaluate the interactions of DGPSGPK and two integrins, including αvβ3 (PDB:1L5G) and α5β1 (PDB:3VI4). A previous study showed that some proteins and peptides can bind to integrin αvβ3 and inhibit cytoskeleton production in osteoclasts, which decreased the differentiation of osteoclasts [47]. Meanwhile, some proteins and peptides can bind to integrin α5β1, and activate the focal adhesion kinase/extracellular signal-regulated kinase (FAK/ERK) pathway. The activation of this pathway increased the expression of phosphorylated runt-related transcription factor-2, which could promote the differentiation of osteoblasts [48]. Furthermore, the peptides binding to integrin α5β1 could enhance the adhesion, proliferation, and differentiation of osteoblasts [49].

**Figure 9:**
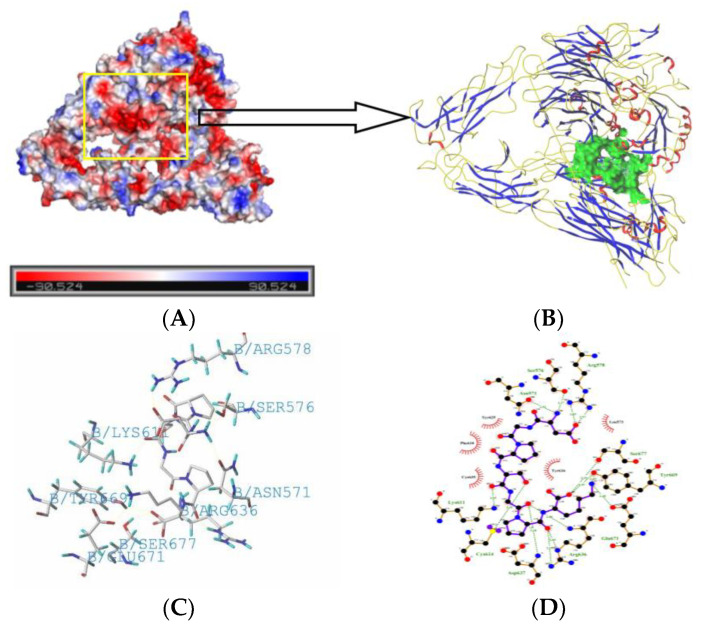

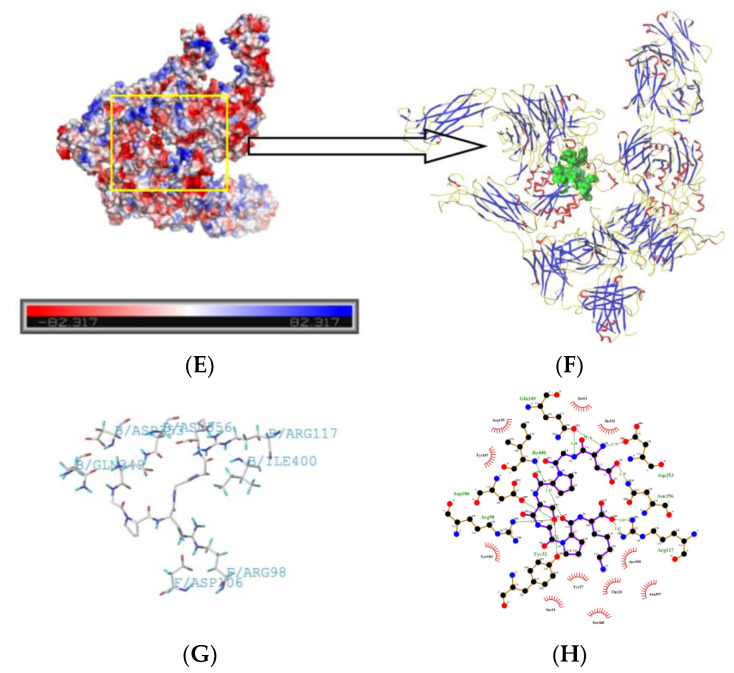
Docking simulation for the interaction of DGPSGPK and integrins: PDB: 1L5G (**A**–**D**) and PDB: 3VI4 (**E**–**H**). (**A**,**E**) surface electrostatic potential energy of the integrator, red is negative voltage, blue is positive voltage; (**B**,**F**) 3D structure of DGPSGPK docked with integrin; (**C**,**G**) interaction sites of DGPSGPK and amino acids in the integrins; (**D**,**H**) 2D diagram of the interaction sites of DGPSGPK and amino acids in the integrins.

In this study, the T-scores obtained for DGPSGPK docking with 1L5G and 3VI4 were 10.50 and 8.24, which indicated that DGPSGPK can strongly interact with αvβ3 and α5β1. As shown in Figure 9, the interactions between DGPSGPK and integrins included electrostatic interactions, hydrogen bonding, and hydrophobic interactions. Electrostatic interactions are interactions in which positive and negative electrons attract or repel each other [50]. As shown in Figure 9A,E, the red ones are positive electron ions and the blue ones are negative electron ions. In the docking pocket, the positive and negative ions are attracted to each other through electrostatic interactions to make the structure stable. It shows that electrostatic interactions play an important role in molecular docking of DGPSGPK with 1L5G and 3VI4. As shown in Figure 9D,H, the number of hydrogen bonds docked between DGPSGPK and integrins were 13 and 10, respectively. Meanwhile, hydrogen bonding interactions of DGPSGPK with 1L5G included Arg578, Ser576, Asn571, Lys611, Cys614, Asp637, Arg636, Glu671, Tyr669, and Ser667, and DGPSGPK with 3VI4 included Gln349, Ile400, Asp 106, Arg98, Tyr32, Arg117, Asn356, and Asp353. In addition, the length of the hydrogen bond predicts the strength of the hydrogen bond. In this study, the bond lengths of the hydrogen bonds are short (2.56–3.35 Å), indicating that hydrogen bonding also plays a significant role in molecular docking between DGPSGPK and integrins. Moreover, previous studies have shown that hydrophobic interactions can effectively improve the stability of docking [51]. As shown in Figure 9D,H, hydrophobic interactions of DGPSGPK with 1L5G included Tyr625, Phe610, Cys635, Tyr634, and Leu573, and with 3VI4 included Arg155, Tyr107, Tyr103, Ser31, Tyr27, Ser360, Thr28, Asn397, Ser399, Ile352, and Ser61. The molecule docking results indicated that DGPSGPK docking with integrins may possess an ability to inhibit osteoclast differentiation and proliferation and promote osteoblast differentiation and proliferation. Our results are similar to the previous study, which showed that peptide YPRKDETGAERT with good osteogenic activity had high interactions with 1L5G and 3VI4 [52].

### 2.6. Effects of DGPSGPK on the RANKL-Induced Osteoclasts

**Figure 10:**
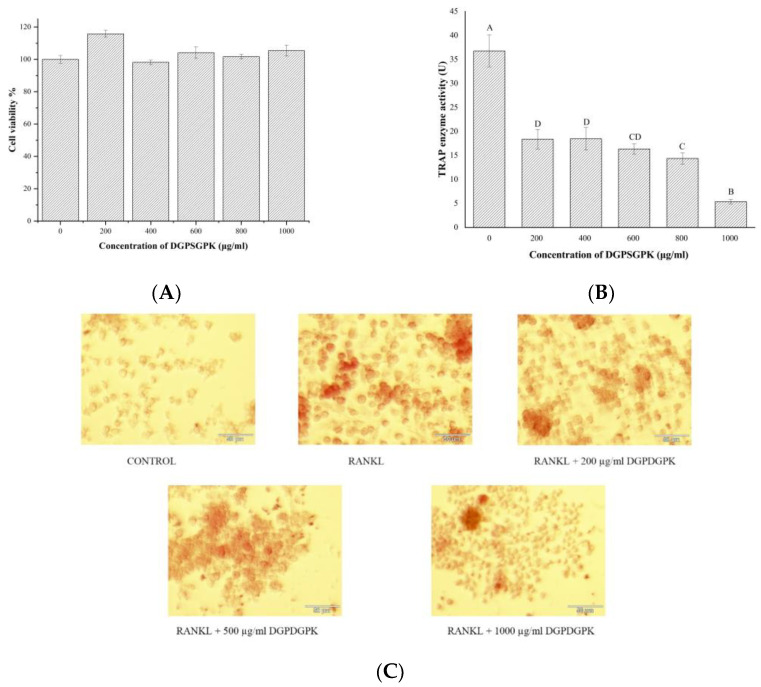
Effects of DGPSGPK with different concentrations on osteoclast differentiation. (**A**) Cell viability. (**B**) TRAP activity. (**C**) TRAP staining.

An MTT assay was used to determine the effects of DGPSGPK with different concentrations on RAW 264.7 cells. As shown in Figure 10A, DGPSGPK with different concentrations (200, 400, 600, 800, 1000 µg/mL) showed no toxicity for RAW 264.7 in this study.

TRAP activity and staining are important indicators to determine the differentiation of RAW 264.7 cells into osteoclasts [53]. In this study, RAW 264.7 cells cultured with RANKL were differentiated into osteoclasts, according to the results of Figure 10B,C. RANKL-induced osteoclasts were co-cultured with DGPSGPK of different concentrations. As shown in Figure 10B, TRAP activity of osteoclasts was dose-dependently reduced by DGPSGPK with different concentrations. This suggests an inhibitory effect of DGPSGPK on the differentiation of osteoclasts. As shown in Figure 10C, we observed a dose-dependent decrease in TRAP enzyme by staining. The staining result was consistent with the results of the TRAP enzyme activity assay. The results showed that DGPSGPK could inhibit the differentiation of osteoclasts. It was accordance to the previous studies. Miyamoto et al. reported that peptide GRGDS could inhibit the proliferation of osteoclasts [54]. Lundberg et al. presented that vasoactive intestinal peptide can inhibit osteoclast activity by binding to receptors in the osteoclast province [55]. Kaneda et al. isolated cyclic peptides–cyclolinopeptides from flaxseed with the ability to inhibit osteoclast differentiation [56].

### 2.7. Effects of DGPSGPK on the Proliferation, Differentiation, and Mineralization of MC3T3-E1 Cells

**Figure 11:**
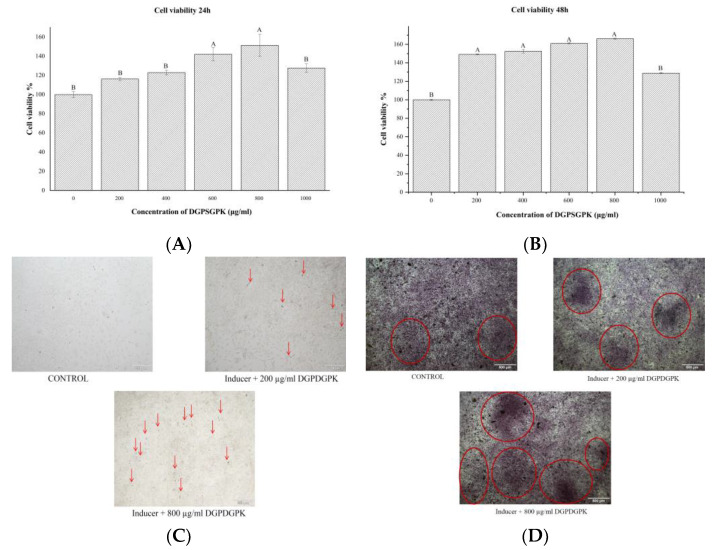
Effects of DGPSGPK with different concentrations on osteoblast proliferation, differentiation, and mineralization. (**A**,**B**) cell viability of MC3T3-E1 cells; (**C**): ALP staining at 7 days; (**D**): MC3T3-E1 mineralization staining at 21 days.

As shown in Figure 11A,B, after co-culture of MC3T3-E1 cells with different concentrations of DGPSGPK for 24 and 48 h, DGPSGPK showed a significant proliferative effect on MC3T3-E1 cells. The proliferative effects of DGPSGPK with a dose of 800 µg/mL at 24 and 48 h on MC3T3-E1 cells reached 142% and 166%, respectively.

MC3T3-E1 cells can differentiate into mature osteoblasts with the culture of β-glycerophosphate and ascorbic acid. ALP enzyme activity is an important indicator for the evaluation of osteoblast differentiation. As shown in Figure 11C, we performed ALP enzyme activity staining on MC3T3-E1 cells cultured for 7 days. We clearly observed that DGPSGPK with 200 and 800 µg/mL could obviously promote the increase in ALP enzyme activity. The staining results indicated that DGPSGPK may promote the differentiation of MC3T3-E1 cells. It was accordance with previous studies. Shi et al. showed that lactoferrin is effective in promoting the differentiation and proliferation of osteoblasts [57]. Deer tendon enzymatic digest protein can regulate the gene expression thus promoting the proliferation of osteoblasts and the formation of cell matrix. Xu et al. [52] and Shi et al. [57] found that both blue mussel and bovine lactoferrin-derived peptides have the ability to inhibit osteoclast differentiation and promote osteoblast proliferation and differentiation.

The bone extracellular matrix matures at the end of differentiation and subsequently mineralizes into bone tissue [58]. As shown in Figure 11D, alizarin red staining of MC3T3-E1 cells cultured for 21 days were performed. It can be observed that DGPSGPK with 200 and 800 µg/mL could promote the production of mineralized significantly and it was in a dose-dependent manner. This result indicated that DGPSGPK can effectively promote the differentiation as well as mineralization of MC3T3-E1.

## 3. Materials and Methods

### 3.1. Materials and Reagents

Tilapia bone was provided from Ocean King Fisheries Co. Ltd. (Yunnan, China). Papain, pepsin, flavoring protease, neutral protease, complex protease, basic protease, and trypsin protease were provided by Shanghai Yuanye Biological Technology Co. Ltd. (Shanghai, China), and animal protease was provided by Pangbo Biological Engineering Co. Ltd. (Nanjing, China). Peptides were produced by Shanghai Synpeptide Co. Ltd. (Shanghai, China). Hydrolytic protease was obtained from Novozymes Biotechnology Co. Ltd. (Beijing, China). Acetonitrile and trifluoroacetic acid (TFA) of mass spectroscopic grade were obtained from Merck KGaA (Darmstadt, Germany). Raw 264.7 and MC3T3-E1 cells were obtained from the Kunming Institute of Zoology cell bank. Tartrate resistant acid phosphatase (TRAP) kit, acid phosphatase (ALP) kit, alizarin red staining kit, and BCA Protein Assay Kit were obtained from Beyotieme Co. Ltd. (Shanghai, China). TRAP/ALP stain kit was obtained from Wako Co. Ltd. (Wako, Japan). All the other chemicals and reagents were of analytical grade.

### 3.2. Preparation of Different Tilapia Bone Enzymatic Hydrolysates

Tilapia bone was washed for 30 min in a continuous water bath at 50 °C and treated under high pressure (121 °C, 20 min). After cooling to room temperature, the tilapia bone was homogenized in a ratio of 1:2 with distilled water. Nine enzymes, namely, papain, flavoring protease, complex protease, neutral protease, pepsin, basic protease, hydrolytic protease, animal protease, and trypsin, were utilized to hydrolyze the tilapia bone. The hydrolysis conditions are shown in Table 1. After hydrolysis, the enzyme was deactivated in a 100 °C water bath for 10 min. The hydrolysates were centrifuged (6000× *g*, 10 min) after cooling to room temperature, and the supernatants were collected and frozen.

### 3.3. Determination of DH

Ninhydrin colorimetry was used to determine the DH in each enzymatic hydrolysate [18], and the content of protein in the hydrolysate was determined by Kjeldahl definition nitrogen method. DH was calculated according to Formula (1).

DH (%) = C/(N × h_tot_) × 100% (1)

where DH is the degree of hydrolysis (%), C is the content of –NH_2_ within the hydrolysates (mmol/L), N is the content of protein in the hydrolysates (mg/mL), and h_tot_ is the total number of peptides bonds per unit weight. The h_tot_ for TBEH was 8.41 mmol per gram of protein.

### 3.4. Calcium-Binding Capacity Assay

The previous method was used in assessing the calcium binding capability of different hydrolysates [59]. Approximately 1 mg of sample was mixed in 1 mL of distilled water and then combined with 2 mL of 5 mmol/L CaCl_2_ solutions. After adjusting the pH to 7.8 with 0.01 mol/L NaOH, the mixture was incubated for 30 min in 37 °C water. Approximately 4 mL of phosphate buffered saline (20 mmol/L, pH 7.8) was mixed, and the mixture was bathed at 37 °C for 30 min. The mixture was then centrifuged for 15 min at 6000× *g*, and the supernatant was obtained. The experimental condition for the blank group was the same as above. The calcium content was determined through flame atomic absorption spectrometry (novAA^®^350, Analytikjena, Germany), and the calcium binding capacity was computed using Formula (2):

Calcium-binding capacity (µg/mg) = (M_1_ − M_0_)/M (2)

where M_1_ is the content of calcium in the supernatant (µg), M_0_ is the content of calcium in the blank solution (µg), and M is the content of protein in the sample (mg).

### 3.5. Calcium-Binding Peptides

The calcium binding capacities of nine different enzymatic hydrolysates were analyzed. The hydrolysate with the highest calcium binding capacity was investigated, noting TBEH. TBEH was separated utilizing an RP-HPLC system and a SHIMADZU C-18 preparation column (20 × 250 mm, Shimadzu, Japan). The mobile phase A was 0.1% TFA in acetonitrile and mobile phase B was 0.1% TFA in water. The elution gradient was 8–75% A for 35 min. The flow rate was 10 mL/min and the absorbance was determined at 220 nm. Each peak fraction was collected, and its calcium binding capacity was determined.

A semi-preparation column SB-C18 ZORBAX (9.4 × 250 mm, Agi-Lent Technologies, Santa Clara, CA, USA) was further used in separating the fraction with the highest calcium binding capacity. The mobile phase A was 0.1% TFA in acetonitrile and mobile phase B was 0.1% TFA in water. The flow rate was 2 mL/min. The elution gradient was 8–35% A for 28 min. The absorbance was determined at a wavelength of 220 nm. Each peak fraction was collected, and calcium binding capacity was determined. The calcium-binding capacity of the fraction with the highest potential was further analyzed.

### 3.6. Identification of Amino Acid Sequence

Ultra-performance liquid chromatography quadrupole orbitrap mass spectrometry (UPLC-Q-Orbitrap-MS^2^, Q-ExActive, Thermo Fisher Scientific, Waltham, MA, USA) was used in evaluating the peptide in the fraction separated with our previous method [60]. *De Novo ^TM^* Software (Peak Studio 7.5, BioInfor-Matics Solutions, Inc. Waterloo, ON, Canada) was used in identifying the peptide’s amino acid sequence and molecular weight. The peptide with a high average local confidence (ALC) value (>85%) was considered for further analysis.

### 3.7. Preparation of Calcium–Peptide Chelate

The chelating process was used in producing the peptide–calcium chelate. In brief, 2 mL of CaCl_2_ (5 mM) was mixed with 1 mL of peptide (1 mg/mL), and the pH was adjusted to 7.8 with 10 mM NaOH. The mixture was bathed at 37 °C for 30 min. Approximately 4 mL of phosphate buffer solution (20 mM, pH 7.8) was added into the mixture at 37 °C for 30 min. The mixture was centrifuged at 6000× *g* for 10 min for the preparation of the calcium–peptide chelate.

### 3.8. Structure Characterization of the Calcium–Peptide Chelate

#### 3.8.1. FTIR

The purified peptide and calcium–peptide chelate (10 mg) were evenly ground with 100 mg of dry KBr. The samples were scanned using a Fourier transform infrared spectrometer (Alpha, Bruker Optics, Ettlingen, Germany) at a scan range from 4000 to 400 cm^−1^, and the absorption spectra were measured.

#### 3.8.2. XRD

The purified peptide and calcium–peptide chelate (10 mg) were ground and placed in the sample area. XRD spectra were recorded with an X-ray diffractometer (Empyrean, Panalytical B.V. Netherlands). The scanning speed was 4°/min and the scanning angle (2θ) was 5°–90°.

#### 3.8.3. UPLC-Q-Orbitrap-MS^2^

UPLC-Q-Orbitrap-MS^2^ was used in evaluating the purified peptide and calcium–peptide chelate [61]. The UPLC conditions were as follows: Hypersil Gold C18 column (2.1 × 100 mm, Thermo Fisher Scientific, USA); mobile phase A, 0.1% formic acid–acetonitrile; mobile phase B, was 0.1% formic acid–water; elution gradient, 5–95% A for 24 min; flow rate, 0.2 mL/min; and injection volume, 2 μL. The absorbance was monitored at 220 nm. The working conditions of the mass spectrometer were as follows: bombardment volume, 20 eV; spray voltage, 3.6 kV; and flow rate, 5 L/min. In positive ion mode, the m/z spectrum from 120 to 1800 was scanned.

#### 3.8.4. Construction of the Possible Molecular Modes

The initial structure of the calcium–peptide chelate was obtained by using the online peptide structure builder PEP-FPLD.

### 3.9. Molecular Docking

Molecular docking was carried out in SYBYL-X 2.0 software. The structures of the peptides were constructed and optimized using a minimization ligand scheme in STBYL-X 2.0 software. Two integrins (PDB: 1L5G and 3VI4) were downloaded in the database. The interaction of DGPSGPK and two integrins was analyzed, and T-score, hydrogen bond, and bond distance were calculated using SYBYL-X 2.0 software. If the T-score score was higher than 6.0, the rustles were accepted. The electrostatic potential energy and secondary structure of the protein were produced using Pymol software and Ligplus software, respectively.

### 3.10. RAW264.7 Cell Culture

RAW264.7 cells were cultured in Dulbecco’s modified Eagle medium (DMEM) containing 10% PBS and 1% penicillin/streptomycin at 37 °C and 5% CO_2_. RAW264.7 cells at logarithmic growth stage were seeded in 96-well plates with 1 × 10^5^ cells/well. The cells were incubated for 24 h to adhere to the wall. The medium was removed and the samples with different concentrations (0, 200, 400, 600, 800, 1000 µg/mL) were added and incubated for 24 h. The cell viability was measured using MTT assay. In brief, the medium was aspirated, 150 µL of MTT was added and incubated for 4 h. The medium was poured out and 150 µL of DMSO was added and shaken for 10 min. The absorbance values were measured at 570 nm using an enzyme marker (Spectra Max M5; Molecular Devices, Sunnyvale, CA, USA).

#### 3.10.1. TRAP Activity Assay

RAW264.7 cells were cultured with 50 ng/mL receptor activator of NF-κB ligand (RANKL) and the medium was replaced every 2 days. After 5 days, RAW 264.7 cells were differentiated into osteoclasts. The cells were cultured with the sample of different concentrations for one day. TRAP activity of the cells was measured using the relative kit.

#### 3.10.2. TRAP Staining

The RANKL-induced osteoclasts were plated in 48-well plates at 1 × 10^5^ cells/well and cultured with the sample with different concentrations of sample (0, 200, 500, 1000 µg/mL) for one day. The cells were stained using the TRAP/ALP stain kit.

### 3.11. MC3T3-E1 Cell Culture and Staining

MC3T3-E1 cells were cultured in α-minimum essential medium (α-MEM) containing 10% PBS and 1% penicillin/streptomycin at 37 °C and 5% CO_2_. MC3T3-E1 cells at logarithmic growth stage were seeded in 96-well plates with 1 × 10^5^ cells/well. The cells were incubated for 24 h to adhere to the wall. The medium was removed and the samples of different concentrations (0, 200, 400, 600, 800, 1000 µg/mL) were added and incubated for 24 and 48 h. The cell viability was measured using MTT assay.

MC3T3-E1 cells were plated in 24-well plates at 2 × 10^5^ cells/well. MC3T3-E1 cells were cultured in medium containing 10 mM β-glycerophosphate, 50 µg/mL ascorbic acid, and the samples of different concentrations (0, 200, 800 µg/mL). The medium was changed every other day. The cells were stained using TRAP/ALP stain on day 7 and alizarin red staining on day 21 using the relative kits.

### 3.12. Statistical Analysis

All examinations were performed three times, and each result was reported as mean ± standard deviation (SD). SPSS statistics software was used in statistical analysis (version 19.0, SPSS Inc., Chicago, IL, USA). A *p* value of <0.05 indicated a significant difference.

## 4. Conclusions

In this study, animal protease was used in obtaining tilapia bone hydrolysate with high calcium binding capacity. The hydrolysate delivered a new calcium chelating peptide, which was identified as DGPSGPK. The structure of DGPSGPK and DGPSGPK-Ca were identified through FTIR, XRD, SEM, and UPLC-Q-Orbitrap-MS^2^. The results indicated that Ca^2+^ was mainly chelated with the carbonyl and nitrogen groups of Lys and Pro residues. Molecular simulation showed that the carbonyl groups of Asp, Pro, Ser, and Lys residues involved the chelating of Ca^2+^ and peptide. Furthermore, DGPSGPK could effectively inhibit osteoclast differentiation and increase osteoblast proliferation, differentiation, and mineralization. This study provided a scientific basis for the preparation of bioactive peptides and calcium-binding peptides that can be used as functional supplements for regulating osteoporosis.

## Figures and Tables

**Table 1:** Hydrolysis temperature, pH, and time of nine proteases.

Types	E/S/%	Temperature/°C	pH	Time/h	Source	Type
Papain	2.5	55	7.0	4	Vegetal	Endopeptidase
Flavoring protease	2.5	55	7.0	4	Microbial	Endo and exopeptidase
Complex protease	2.5	55	6.0	4	Microbial	Endopeptidase
Neutral protease	2.5	50	7.0	4	Microbial	Endopeptidase
Basic protease	2.5	55	9.0	4	Microbial	Endopeptidase
Pepsin	2.5	37	2.0	4	Animal	Endopeptidase
Hydrolytic protease	2.5	60	7.0	4	Microbial	Endopeptidase
Trypsin	2.5	37	8.0	4	Animal	Endopeptidase
Animal protease	2.5	50	7.0	4	Animal	Endo and exopeptidase

## Data Availability

Not applicable.

## References

[1] Hou T., Wang C., Ma Z., Lui W., He H. (2015). Desalted duck egg white peptides: Promotion of calcium uptake and structure characterization. J. Agric. Food Chem..

[2] Shin H.L., Jung I.Y., Suck M.H., Dae H.H., Shin Y.L., Il H.K., Sang Y.C. (2005). Phosphorylation of peptides derived from isolated soybean protein: Effects on calcium binding, solubility and influx into Caco-2 cells. BioFactors.

[3] Caroline M.G. (2021). Calcium-sensing receptor signaling-How human disease informs biology. Curr. Opin. Endocr. Metab. Res..

[4] Giovianni B., Jonathan O.R., Alan L.K., James A.O. (2020). Physicochemical and bulk handling properties of micronised calcium salts and their application in calcium fortification of whey protein-based solutions. J. Food Eng..

[5] Guo L., Harnedy P.A., Li B., Hou H., Zhang Z., Zhao X., Fitzgerald R.J. (2014). Food protein-derived chelating peptides: Biofunctional ingredients for dietary mineral bioavailability enhancement. Trends Food Sci. Technol..

[6] Martina V., Leif H.S. (2014). Calcium nutrition. bioavailability and fortification. LWT Food Sci. Technol..

[7] Zhang X.W., Qi J., Li M.Y., Liu H.P., Wang Q., Wu Y.R., Niu L.L., Liu Z.T. (2021). Isolation of a novel calcium-binding peptide from phosvitin hydrolysates and the study of its calcium chelation mechanism. Food Res. Int..

[8] Wu W., He L., Li C., Zhao S., Liang Y., Yang F., Zhang M., Jing G., Ma M. (2019). Phosphorylation of porcine bone collagen peptide to improve its calcium chelating capacity and its effect on promoting the proliferation, differentiation and mineralization of osteoblastic MC3T3-E1 cells. J. Funct. Foods.

[9] Shunichi Y., Gen M., Tomohiro S., Tomoka H., Taku E., Daisuke T., Cai H., Yuan T., Hend A., Masahiko T. (2021). Cardiotrophin Like Cytokine Factor 1 (CLCF1) alleviates bone loss in osteoporosis mouse models by suppressing osteoclast differentiation through activating interferon signaling and repressing the nuclear factor-κB signaling pathway. Bone.

[10] Wang Q., Shen X., Chen Y., Chen J., Li Y. (2021). Osteoblasts-derived exosomes regulate osteoclast differentiation through miR-503-3p/Hpse axis. Acta Histochem..

[11] Qing X.B., Wen K., Wu X.X., Yao Z. (2021). MiR-183 regulates the differentiation of osteoblasts in the development of osteoporosis by targeting Smad4. Acta Histochem..

[12] Zu Y., Liang X.D., Du J., Zhou S., Yang C. (2015). Binding of integrin α1 to bone morphogenetic protein receptor IA suggests a novel role of integrin α1β1 in bone morphogenetic protein 2 signalling. J. Biomech..

[13] Zuzana S., Carole L.H., Sofia A., Caroline M., Sophie D.N., Pascal S., Pierre J.M. (2015). Wnt/β-catenin signaling mediates osteoblast differentiation triggered by peptide-induced α5β1 integrin priming in mesenchymal skeletal cells. J. Biol. Chem..

[14] Polong W., Shaochen L., Chiachen C., Seiji M., Nobuaki A., Wenguey W., Yoshikazu T. (2006). Non-cytotoxic cobra cardiotoxin A5 binds to αvβ3 integrin and inhibits bone resorption. J. Biol. Chem..

[15] Weilin S., Matsui T. (2017). Current knowledge of intestinal absorption of bioactive peptides. Food Funct..

[16] Chen D., Mu X., Huang H., Nie R., Liu Z., Zeng M. (2014). Isolation of a calcium-binding peptide from tilapia scale protein hydrolysate and its calcium bioavailability in rats. J. Funct. Foods.

[17] Charoenphun N., Cheirsilp B., Sirinupong N., Youravong W. (2013). Calcium-binding peptides derived from tilapia (*Oreochromis niloticus*) protein hydrolysate. Eur. Food Res. Technol..

[18] Liu B.T., Zhuang Y.L., Sun L.P. (2019). Identification and characterization of the peptides with calcium-binding capacity from tilapia (*Oreochromis niloticus*) skin gelatin enzymatic hydrolysates. J. Food Sci..

[19] Shun L.H., Li N.Z., Xixi C., Shaoyun W., Yifan H., Jing H., Pingfan R. (2015). Purification and characterisation of a glutamic acid-containing peptide with calcium-binding capacity from whey protein hydrolysate. J. Dairy Res..

[20] Xu Z., Chen H., Wang Z., Fan F., Shi P., Tu M., Du M. (2019). Isolation and characterization of peptides from *Mytilus edulis* with osteogenic activity in mouse MC3T3-E1 preosteoblast cells. Agric. Food Chem..

[21] Yuan Y., Dai Y., Zhang Z., Gong Y., Yuan Y. (2020). Technical efficiency of different farm sizes for tilapia farming in China. Aquac. Res..

[22] Lin S., Hu X., Li L., Yang X., Chen S., Wu Y., Yang S. (2021). Preparation, purification and identification of iron-chelating peptides derived from tilapia (*Oreochromis niloticus*) skin collagen and characterization of the peptide-iron complexes. LWT Food Sci. Technol..

[23] Chuesiang P., Sanguandeekul R. (2015). Protein hydrolysate from tilapia frame: Antioxidant and angiotensin I converting enzyme inhibitor properties. Int. J. Food Sci. Technol..

[24] Liao W., Chen H., Jin W., Yang Z., Cao Y., Miao J. (2020). Three newly isolated calcium-chelating peptides from tilapia bone collagen hydrolysate enhance calcium absorption activity in intestinal caco-2 cells. J. Agric. Food Chem..

[25] Zhuang Y.L., Sun L.P. (2011). Preparation of reactive oxygen scavenging peptides from tilapia (*Oreochromis niloticus*) skin gelatin: Optimization using response surface methodology. J. Food Sci..

[26] Sun L.P., Zhuang Y.L. (2013). Antiphotoaging effect and purification of an antioxidant peptide from tilapia (*Oreochromis niloticus*) gelatin peptides. J. Funct. Foods.

[27] Zhao T., Liu B.T., Yuan L., Sun L.P., Zhuang Y.L. (2019). ACE inhibitory activity in vitro and antihypertensive effect in vivo of LSGYGP and its transepithelial transport by Caco-2 cell monolayer. J. Funct. Foods.

[28] Clemente A. (2001). Enzymatic protein hydrolysates in human nutrition. Trends Food Sci. Technol..

[29] Beaubier S., Framboisier X., Ioannou I., Galet O., Kapel R. (2018). Simultaneous quantification of the degree of hydrolysis, protein conversion rate and mean molar weight of peptides released in the course of enzymatic proteolysis. J. Chromatogr. B.

[30] Sandroddin S.G., Georges G. (2002). Theory of optimization of the experimental conditions of preparative elution chromatography: Optimization of the column efficiency. Anal. Chem..

[31] Yanlan L., Xixi C., Xiaoping W., Shengnan L., Shaoyun W. (2019). Fabrication of snapper fish scales protein hydrolysate-calcium complex and the promotion in calcium cellular uptake. J. Funct. Foods.

[32] Wayne A.H., Jerme K. (1973). Carp muscle calcium-binding protein. J. Biol. Chem..

[33] Wu W., Li B., Hu H., Zhang H., Xue Z. (2017). Isolation and identification of calcium-chelating peptides from pacific cod skin gelatin and their binding properties with calcium. Food Funct..

[34] Cui P., Lin S., Jin Z., Zhu B., Liang S., Sun N. (2018). In vitro digestion profile and calcium absorption studies of a sea cucumber ovum derived heptapeptide-calcium complex. Food Funct..

[35] Zhang L., Lin Y., Wang S. (2018). Purification of algal calcium-chelating peptide and its physical chemical properties. J. Aquat. Food Prod. Technol..

[36] Li W., Ding Y., Zhang X., Li Y., Chen Z. (2017). Isolation of a novel calcium-binding peptide from wheat germ protein hydrolysates and the prediction for its mechanism of combination. Food Chem..

[37] Wu W., He L., Liang Y., Yue L., Peng W., Jin G., Ma M. (2019). Preparation process optimization of pig bone collagen peptide-calcium chelate using response surface methodology and its structural characterization and stability analysis. Food Chem..

[38] Zhao L., Huang S., Cai X., Hong J., Wang S. (2014). A specific peptide with calcium chelating capacity isolated from whey protein hydrolysate. J. Funct. Foods.

[39] Zhang K., Li J., Hou H., Zhang H., Li B. (2019). Purification and characterization of a novel calcium-biding decapeptide from pacific cod (*Gadus macrocephalus*) bone: Molecular properties and calcium chelating modes. J. Funct. Foods.

[40] Cai X., Yang Q., Lin J., Fu N., Wang S. (2017). A specific peptide with calcium-binding capacity from defatted *Schizochytrium* sp. protein hydrolysates and the molecular properties. Molecules.

[41] Xu W., Gao A., Yue C., Zhang X., Li S., Ye C. (2017). Preparation of cucumber seed peptide-calcium chelate by liquid state fermentation and its characterization. Food Chem..

[42] Sun X.D., Ruan S.Y., Zhuang Y.L., Sun L.P. (2021). Anti-osteoporosis effect and purification of peptides with high calcium-binding capacity from walnut protein hydrolysate. Food Funct..

[43] Wang X., Zhang Z., Xu H., Li X., Hao X. (2020). Preparation of sheep bone collagen peptide-calcium chelate using enzymolysis-fermentation methodology and its structural characterization and stability analysis. RSC Adv..

[44] Tanaka M., Hong S.M., Akiyama S., Hu Q.Q., Matsui T. (2015). Visualized absorption of anti-atherosclerotic dipeptide, Trp-His, in Sprague-Dawley rats by LC-MS and MALDI-MS imaging analyses. Mol. Nutr. Food Res..

[45] Shen Y., Maupetit J., Derreumaux P., Tuffery P. (2014). Improved PEP-FOLD approach for peptide and miniprotein structure prediction. J. Chem. Theory Comput..

[46] Ruan S.Y., Sun L.P., He J.L., Zhuang Y.L. (2021). Novel umami peptides from tilapia lower jaw and molecular docking to the taste receptor T1R1/T1R3. Food Chem..

[47] Tzu L., Rong Y., Huang J.T., Liou H.C., Lin Y.M., Woiejer C., Fu W.M. (2017). Inhibition of osteoporosis by the αvβ3 integrin antagonist of rhodostomin variants. Eur. J. Pharmacol..

[48] Vermont P.D., Elvira G.M. (2011). Lunasin potentiates the effect of oxaliplatin preventing outgrowth of colon cancer metastasis, binds to α5β1 integrin and suppresses FAK/ERK/NF-κB signaling. Cancer Lett..

[49] Roberta F., Florian R., Stefanie N., Jose M.M., Javier G., Horst K., Carlos M.M. (2015). Mimicking bone extracellular matrix: Integrin-binding peptidomimetics enhance osteoblast-like cells adhesion, proliferation, and differentiation on titanium. Colloids Surf. B Biointerfaces.

[50] Bondžić A.M., Senćanski M.V., Nikezić A.V.V., Kirillova M.V., André V., Kirillov A.M., Bondžić B.P. (2020). Aminoalcoholate-driven tetracopper(II) cores as dual acetyl and butyrylcholinesterase inhibitors: Experimental and theoretical elucidation of mechanism of action. J. Inorg. Biochem..

[51] Dawei J., Min X., Chibuike C.U., Domonic A. (2020). Physicochemical characterisation, molecular docking, and drug-likeness evaluation of hypotensive peptides encrypted in flaxseed proteome. Curr. Res. Food Sci..

[52] Xu Z., Chen H., Fan F., Shi P., Tu M., Cheng S., Wang Z., Du M. (2019). Bone formation activity of an osteogenic dodecapeptide from blue mussels (*Mytilus edulis*). Food Funct..

[53] Daye L., Wan K.K., Seong J.K., In B.H., Je B.H., Seung H.S., Seil S. (2021). Inhibitory effects of gold and silver nanoparticles on the differentiation into osteoclasts in vitro. Pharmaceutics.

[54] Takeshi M., Fumio A., Osamu O., Katsumasa T., Dirk A., Toshio S. (2000). An adherent condition is required for formation of multinuclear osteoclasts in the presence of macrophage colony-stimulating factor and receptor activator of nuclear factor κB ligand. Blood.

[55] Lundberg P., Lie A., Bjurholm A., Lehenkari P.P., Horton M.A., Lerner U.H., Ransjo M. (2000). Vasoactive intestinal peptide regulates osteoclast activity via specific binding sites on both osteoclasts and osteoblasts. Bone.

[56] Kaneda T., Yoshida H., Nakajima Y., Toishi M., Nugroho A.E., Morita H. (2016). Cyclolinopeptides, cyclic peptides from flaxseed with osteoclast differentiation inhibitory activity. Bioorganic Med. Chem. Lett..

[57] Shi P., Fan F., Chen H., Xu Z., Cheng S., Lu W.L., Du M. (2020). A bovine lactoferrin-derived peptide induced osteogenesis via regulation of osteoblast proliferation and differentiation. J. Dairy Sci..

[58] Yuki T., Masashi K., Kiyoko O.G., Shunji H., Noriko F. (2018). Collagen-derived X-Hyp-Gly-type tripeptides promote differentiation of MC3T3-E1 pre-osteoblasts. J. Funct. Foods.

[59] Liu B.T., Zhuang Y.L. (2019). Structural characterization of peptide calcium chelate VGLPNSR-Ca and its calcium absorption ability in Caco-2 cell monolayer. Chem. J. Chin. Univ.-Chin..

[60] Yuan L., Sun L.P., Zhuang Y.L. (2018). Preparation and identification of novel inhibitory angiotensin-I-converting enzyme peptides from tilapia skin gelatin hydrolysates: Inhibition kinetics and molecular docking. Food Funct..

[61] Hong S.M., Tanaka M., Koyanagi R., Shen W., Matsui T. (2016). Structural design of oligopeptides for intestinal transport model. J. Agric. Food Chem..

